# Ultrasound-Guided Percutaneous Versus Open A1 Pulley Release for Trigger Finger: A Randomized Controlled Trial

**DOI:** 10.3390/jcm14197064

**Published:** 2025-10-07

**Authors:** Süleyman Kaan Öner, Nihat Demirhan Demirkiran, Turan Cihan Dulgeroglu, Sabit Numan Kuyubasi, Suleyman Kozlu, Selçuk Yılmaz

**Affiliations:** 1 Department of Orthopaedics and Traumatology, Kutahya Health Sciences University, Kutahya City Hospital, 43600 Kutahya, Turkey; drdemirhandemirkiran@gmail.com (N.D.D.); tcdulgeroglu@gmail.com (T.C.D.); s.numankuyubasi@hotmail.com (S.N.K.); kozlu_91@hotmail.com (S.K.); 2 Department of Orthopaedics and Traumatology, Antalya City Hospital, 07080 Antalya, Turkey; dryilmazselcuk@gmail.com

**Keywords:** trigger finger, percutaneous release, ultrasound guidance, A1 pulley, open surgery, grip strength

## Abstract

**Background/Objectives**: Trigger finger is a common cause of hand pain and functional limitation. Although open A1 pulley release remains the standard surgical treatment, ultrasound-guided percutaneous needle release (UGPR) has emerged as a minimally invasive alternative. This study aimed to compare the clinical effectiveness and safety of UGPR with open surgery. **Methods**: In this prospective, randomized controlled trial, 146 patients with Green stage 2–4 trigger finger were randomly assigned to UGPR (*n* = 75) or open release (*n* = 71). Pain (VAS), functional status (QuickDASH), and symptom severity (Nirschl Phase Rating) were assessed preoperatively and at postoperative day 3, 1, 6, and 12 months. Grip strength was measured with a digital pinchmeter, and ultrasonographic evaluation of A1 pulley and flexor tendon thickness was performed preoperatively and at 12 months. Subgroup analyses were conducted to address the imbalance in thumb distribution. **Results**: Both groups showed significant postoperative improvements in VAS, QuickDASH, and Nirschl scores (*p* < 0.05 for intragroup comparisons), with no significant differences between groups at 12 months (*p* > 0.05). At the one-year follow-up, grip strength was significantly greater in the UGPR group (*p* = 0.008). Ultrasonographic evaluation revealed greater MCP tendon thickness in UGPR, without clinical impact. Subgroup analyses confirmed comparable functional outcomes in thumb-only and non-thumb cases. Four revisions occurred in the UGPR group (incomplete release, recurrent tenosynovitis, flexor tendon rupture, and neurovascular injury), while none were observed in the open group. **Conclusions**: UGPR and open release provide comparable long-term outcomes in the treatment of trigger finger. UGPR offers the advantages of being minimally invasive and preserving grip strength, although it carries a small risk of incomplete release and procedure-related complications. Patient preference, surgeon expertise, and digit type should guide treatment selection.

## 1. Introduction

Trigger finger (stenosing tenosynovitis) is a common condition resulting from a mismatch between the diameter of the flexor tendons and the surrounding fibro-osseous sheath, particularly at the level of the A1 pulley. This mismatch causes impaired tendon gliding and painful locking or compression of the finger during flexion and extension [1,2]. If left untreated, flexion contractures of the proximal interphalangeal joint can occur [3].

This condition is particularly common in diabetic patients over the age of 30 and is approximately five times more common in diabetic patients than in the general population [4]. It is also more common in women and most commonly occurs in the fifth and sixth decades of life. While the thumb is most affected, other fingers can also be affected [1,4]. The thumb is frequently involved, followed by the ring, middle, and little fingers, and then multiple finger involvement followed by the index finger [1,2].

Initial treatment is usually conservative, involving splinting, nonsteroidal anti-inflammatory drugs (NSAIDs), and corticosteroid injections. However, more advanced, or persistent cases often require surgical intervention, and the traditional approach is open A1 pulley release. First described by Lorthioir in 1958, percutaneous release offers the advantages of faster recovery, less scarring, and outpatient surgery [5,6].

Ultrasound-guided surgery has recently gained popularity due to its ability to visualize anatomical structures dynamically and safely during percutaneous release procedures. This technique potentially minimizes complications such as nerve or tendon injury and allows for real-time confirmation of complete release. Reported success rates for ultrasound-guided percutaneous release range from 90% to 100% [7,8].

In this context, we aimed to conduct a prospective, randomized clinical trial comparing ultrasound-guided percutaneous A1 pulley release with conventional open surgery, focusing on clinical outcomes and functional recovery.

## 2. Materials and Methods

### 2.1. Study Design and Ethical Approval

This prospective, randomized study was conducted at Kütahya Health Sciences University, Department of Orthopedics and Traumatology, following approval by the Clinical Research Ethics Committee (Decision No: 2022.14.01, Date: 5 October 2022). Sample size calculation was performed using G*Power (v3.1.9.7). For a large effect size (d = 0.8), a power of 95%, and a two-tailed alpha of 0.05, the required sample size was calculated as 42 patients per group (total *n* = 84).

### 2.2. Patient Selection

A total of 170 patients diagnosed with stage 2–4 trigger finger according to Green’s classification were included. Patients were randomly assigned into two groups using a web-based randomization tool (https://www.sealedenvelope.com, accessed on 6 September 2025). However, 24 patients were excluded due to loss to follow-up during the postoperative observation period. Consequently, 146 patients completed the study and were included in the final analysis.

In addition to the primary analyses, subgroup analyses were performed to evaluate potential bias due to the disproportionate distribution of thumb cases between groups. Patients were stratified into thumb-only and non-thumb subgroups, and outcomes were compared separately between UGPR and open surgery. Mann–Whitney U tests were applied for between-group comparisons, and medians with interquartile ranges (IQR) were reported. These subgroup analyses were conducted to assess the robustness of the main findings.

Inclusion Criteria:

- Age ≥ 18 years.
- Diagnosis of according to Green’s classification stage 2–4 trigger finger.
- Symptoms lasting ≥3 months.
- No prior treatment (including corticosteroid injections, splinting, physiotherapy, or surgery) for the affected finger.

Exclusion Criteria:

- According to Green’s classification stage 1 trigger finger.
- Previous surgery for trigger finger in the same region.
- Rheumatoid arthritis, osteoarthritis, or De Quervain’s tenosynovitis, Dupuytren’s disease.
- Bony deformities.
- Pregnancy.

### 2.3. Preoperative Evaluation

Preoperative assessments included the Visual Analog Scale (VAS), Quick Disabilities of the Arm, Shoulder, and Hand (QuickDASH), and Nirschl Phase Rating Scale (NPRS). Pinch strength was measured using a portable mechanical pinchmeter (Baseline, Irvington, NY, USA) in accordance with the American Society of Hand Therapists (ASHT) protocol. Patients were seated with the arm adducted, the elbow flexed at 90°, and the forearm and wrist in a neutral position. During measurement, the pulp of the involved finger and the thumb were positioned on opposing surfaces of the device. Patients were instructed to apply maximal force. Three consecutive measurements were taken, and the mean value in kilograms (kg) was recorded for analysis. Ultrasonographic (USG) measurements of A1 pulley thickness and sagittal tendon thickness were performed using a 7–15 MHz linear transducer (GE Logic P5 system (GE Healthcare, Chicago, IL, USA)). All ultrasonographic assessments were performed by the operating surgeon, who had prior structured training and experience in musculoskeletal ultrasonography, ensuring adequate expertise for ultrasound-guided procedures.

### 2.4. Surgical Techniques Open Release

Performed under local anesthesia in the operating room. A transverse (for thumb) or longitudinal (for other digits) 1.5–2 cm palmar incision was made directly over the A1 pulley. The pulley was visualized and released under direct vision. Wound closure was performed using interrupted sutures. A structured rehabilitation program, including tendon gliding and strengthening exercises, was initiated postoperatively.

### 2.5. Ultrasound-Guided Percutaneous Release

**Figure 1:**
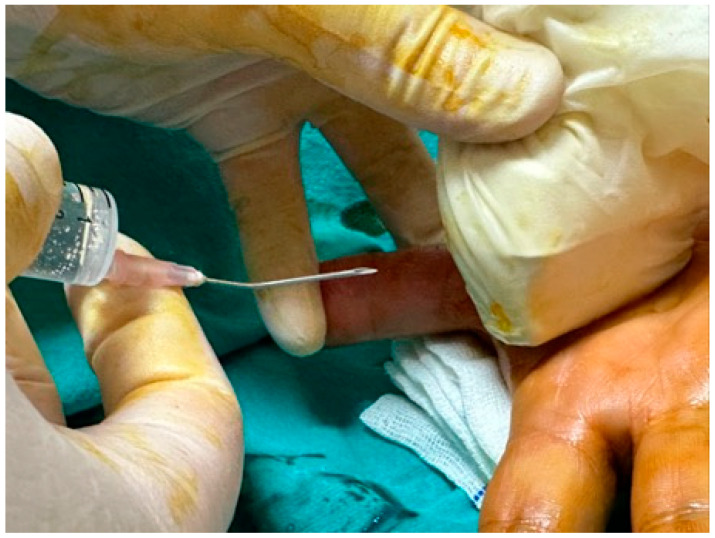
USG-guided percutaneous release technique from the distal of the A1 pulley with an 18 G needle bent at 30 degrees from the middle 1/2.

**Figure 2:**
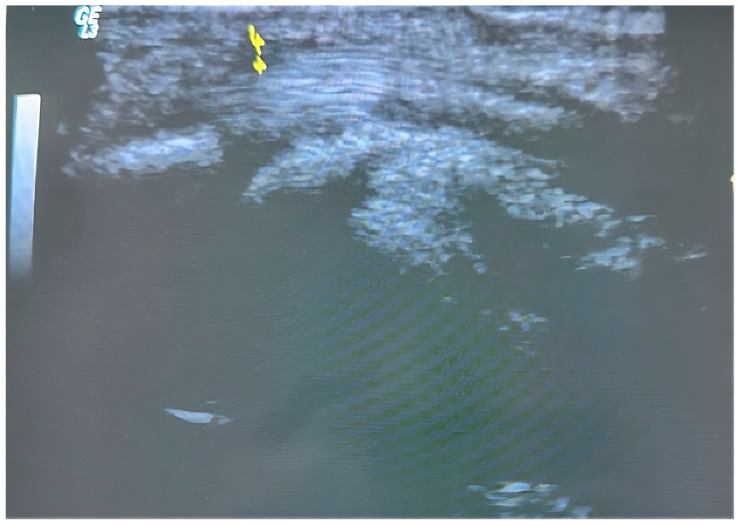
Visualization and measurement of the A1 pulley thickness before percutaneous release. The region indicated by yellow arrows represents the thickest part of the pulley.

**Figure 3:**
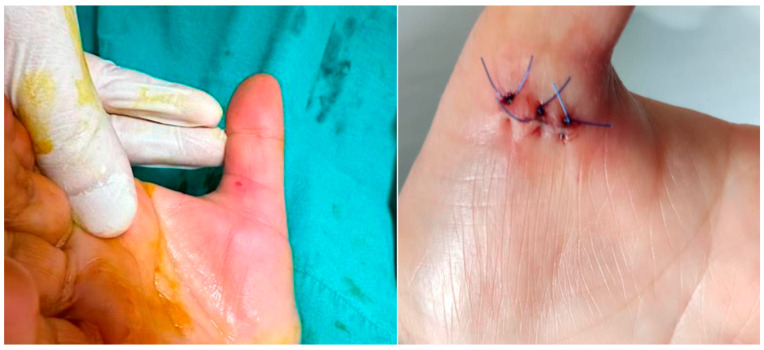
Postoperative scar comparison: The **left** image shows the minimal scar following ultrasound-guided percutaneous release for trigger thumb, while the **right** image displays the surgical scar after conventional open release.

The procedure was performed in an outpatient setting under sterile conditions. After ultrasonographic visualization of the A1 pulley, an 18-gauge needle previously bent to approximately 30 degrees was inserted from the distal edge of the pulley (Figure 1). Through the same needle, 2 cc of prilocaine hydrochloride was administered for local anesthesia. The needle was not withdrawn after injection; instead, it was used to perform the percutaneous release maneuver under continuous real-time ultrasound guidance (Figure 2) (Figure S1). The release was repeated until the triggering sensation and the characteristic clicking sound disappeared, confirming complete release. Compared to open surgery, ultrasound-guided percutaneous release technique required no skin sutures (Figure 3). Post-procedure rehabilitation was identical to that of the open surgery group.

### 2.6. Postoperative Follow-Up

Patients were evaluated at postoperative day 3, and at 1, 6, and 12 months. VAS, QuickDASH, and NPRS scores were recorded at each visit. At the 12-month follow-up, repeat pinch strength and ultrasonographic measurements of A1 pulley and tendon thickness were performed.

### 2.7. Statistical Analysis

Data were analyzed using Jamovi (v2.3.28.0) and JASP (v0.17.2.1). Parametric and non-parametric tests (*t*-test, Mann–Whitney U, Chi-square, Fisher’s exact test, Friedman test, Durbin–Conover post hoc) were applied as appropriate. A *p*-value < 0.05 was considered statistically significant.

## 3. Results

A total of 146 patients were included in the study, of whom 75 underwent ultrasound-guided percutaneous release (UGPR) and 71 underwent open surgery. The mean age was 58.3 ± 9.6 years, and 74.7% of the patients were female. The distribution of affected digits revealed the thumb as the most involved (45.9%). The majority of the cases were stage 2 according to Green’s classification (62.3%) (Table 1).

There were no statistically significant differences between the two groups in terms of age, sex, affected side, Green stage, complication rate, or need for revision surgery (*p* > 0.05) (Table 1).

### 3.1. Functional Scores

- QuickDASH scores were significantly higher in the UGPR group on postoperative day 3 and at the first month (*p* < 0.05), but scores were comparable between groups at 6 and 12 months (Table 2).
- VAS and Nirschl Phase Rating Scale (NPRS) scores followed a similar trend: higher in the early period in the UGPR group, but no significant difference at the final follow-up (*p* > 0.05) (Table 2).

### 3.2. Grip Strength

- At 12 months, the pinch strength values measured with a digital pinchmeter were significantly higher in the UGPR group compared to the open surgery group (*p* < 0.05), indicating better long-term functional recovery (Table 3).
- Ultrasonographic Measurements.
- The increase in flexor tendon thickness at the metacarpophalangeal (MCP) joint level was significantly greater in the open surgery group (*p* < 0.05).
- No significant difference was observed between groups in A1 pulley thickness at 12 months (Table 4).

### 3.3. Complications

- A total of 6 patients (4.1%) experienced complications: 5 in the open surgery group and 1 in the UGPR group. Complications such as incomplete release occurred in 1 patient from the UGPR group. Additionally, revision surgery was required in 4 patients of the UGPR group due to persistent or recurrent triggering, which we reported separately from complications, but this difference did not reach statistical significance (*p* > 0.05).
- In the UGPR group, four complications/revisions were observed, while none occurred in the open surgery group. One stage 3 thumb case developed a flexor tendon rupture, identified at the first postoperative week, which required surgical repair. Two stage 3 patients experienced recurrent pain and tenosynovitis; one was managed with re-operation using open release, and the other with conservative treatment including steroid injection and physiotherapy. In one patient, digital artery and nerve injury occurred intraoperatively. All patients recovered satisfactorily, although mild residual paresthesia persisted in the neurovascular injury case. No revisions or major complications were noted in the open group.

Subgroup analyses addressing the imbalance in thumb distribution showed that UGPR and open release yielded comparable results for QuickDASH, VAS, and pinch strength at 12 months in both thumb-only and non-thumb cases (Supplementary Tables S1 and S2). The only significant difference was a slightly greater MCP tendon thickness in the UGPR group, which did not affect functional outcomes.

## 4. Discussion

This study prospectively compared ultrasound-guided percutaneous release (UGPR) and conventional open surgery for the treatment of trigger finger, focusing on clinical outcomes, functional recovery, and structural changes. Our results demonstrate that both interventions yield comparable efficacy in terms of long-term pain relief and functional improvement, while each approach possesses distinct short-term advantages and limitations.

Patients treated with UGPR experienced significantly higher pain scores (VAS and NPRS) in the early postoperative period (day 3 and 1st month) compared to those treated with open surgery. This finding may be attributed to several factors. First, percutaneous release, despite being minimally invasive, can induce localized tissue irritation due to multiple blind or semi-blind attempts at pulley division, especially during the operator’s early learning curve. Second, incomplete decompression in the early healing phase might delay symptom resolution. Previous studies have similarly reported slightly increased discomfort with percutaneous release techniques in the short term, although pain levels equalize in the long run [9,10]. Similarly, the transient differences observed at the three-month follow-up may be explained by postoperative inflammatory changes and temporary tendon gliding resistance in the UGPR group, whereas open release ensures immediate and complete decompression. These differences diminished by 6 and 12 months, confirming the comparable long-term efficacy of both techniques.

While open surgery showed slightly better QuickDASH scores in the early postoperative period, both groups demonstrated statistically similar functional outcomes by the 6th and 12th months. These findings are consistent with prior randomized studies showing equivalent mid- to long-term improvements in hand function with both techniques [11,12]. Notably, the standardized postoperative rehabilitation protocol employed for all patients may have contributed to these favorable and uniform results.

One of the most compelling findings of our study was the significantly higher pinch strength at 12 months in the UGPR group. This may be due to the preservation of surrounding soft tissue structures, reduced postoperative inflammation, and absence of scar-related adhesions. Open surgery, although precise, involves a skin incision, soft tissue dissection, and suture placement, all of which can trigger fibrosis, leading to tendon gliding resistance and impaired force transmission [10,13]. This phenomenon is supported by biomechanical models and cadaveric studies demonstrating that even subtle disruptions in the fibro-osseous tunnel architecture can affect flexor tendon efficiency [14].

Ultrasonographic evaluation revealed a significantly greater increase in MCP flexor tendon thickness in the open surgery group. This may reflect reactive hyperplasia, tenosynovitis, or compensatory hypertrophy due to altered tendon mechanics [15]. Conversely, the absence of a significant difference in A1 pulley thickness at 12 months suggests that both techniques achieve comparable decompression and structural remodeling. Recent imaging studies have shown that pulley thickness correlates with symptom severity and may serve as a prognostic marker in trigger finger management [16].

The overall complication rate was low in both groups. However, all four cases requiring revision surgery were initially treated with UGPR. This underscores a known limitation of percutaneous techniques, namely the risk of incomplete release. In the absence of direct visualization, ensuring full division of the A1 pulley can be challenging. Nevertheless, our use of high-frequency ultrasound enabled real-time confirmation of pulley disruption and likely mitigated risks associated with neurovascular injury, which is a major concern in blind percutaneous techniques. Similar studies in the literature also highlight the safety advantages of ultrasound guidance in preventing radial and ulnar digital nerve injury [17,18]. In addition, the presence of pre-existing conditions such as diabetes may predispose patients to higher complication and revision rates. Although our study was not sufficiently powered to establish statistical significance, this observation is consistent with previous reports highlighting impaired tendon healing and increased adhesion risk in diabetic patients [4].

Our findings support the growing role of ultrasound-guided techniques in hand surgery. UGPR offers several logistical and clinical advantages: it can be performed in an outpatient setting under local anesthesia, reduces operative time, minimizes postoperative immobilization, and facilitates faster return to daily activities [1,19,20]. Importantly, these benefits do not compromise long—term efficacy. In carefully selected patients, especially those with isolated A1 pulley involvement and no prior interventions, UGPR may be considered a first-line option.

However, open surgery remains a reliable choice, especially in cases with advanced disease (e.g., Green stage 4), recurrent trigger finger, or unclear anatomical landmarks. The surgeon’s familiarity with both techniques and access to high resolution ultrasound are also crucial considerations [21].

As Chan et al. emphasize, the use of ultrasound imaging in hand and wrist surgery is operator-dependent and requires a significant learning curve. Increased surgeon experience has been shown to be directly correlated with increased diagnostic accuracy and procedural success [22]. This observation is consistent with our own clinical experience, where procedural success rates gradually improve as surgeons gain more ultrasound experience.

In this randomized controlled study, both UGPR and open surgery demonstrated comparable efficacy in relieving pain and improving functional outcomes in patients with trigger finger. While early postoperative discomfort was slightly greater in the UGPR group, no long-term differences were observed in symptom or disability scores. Importantly, patients who underwent UGPR exhibited significantly greater grip strength at the one-year follow-up, suggesting enhanced functional recovery associated with the minimally invasive nature of the procedure [23,24].

Although randomization was applied, an imbalance occurred with a higher proportion of thumbs in the UGPR group (58.7%) compared with the open group (32.4%). Because thumb cases are known to be technically easier and recover faster, this imbalance could represent a potential source of bias. To address this, we performed subgroup analyses by digit type. Both thumb-only and non-thumb analyses confirmed that the functional and pain outcomes were similar between groups, thereby supporting the robustness of our main conclusions. The only difference observed was greater MCP tendon thickness in the UGPR group, without functional relevance.

In conclusion, this prospective randomized study demonstrates that UGPR and conventional open surgery are comparably effective in achieving long-term symptom relief and functional improvement in patients with trigger finger. Although UGPR causes slightly more discomfort in the early postoperative period, it offers significant advantages in preserving grip strength and minimizing long-term soft tissue disruption. With the additional advantages of lower procedural costs, shorter operative times, outpatient applicability, and elimination of suture requirements, UGPR represents a safe, cost-effective, and patient-friendly alternative treatment method.

## 5. Limitations

This study has several limitations. First, the procedures were performed by a single surgeon, which may limit generalizability. The follow-up period of 12 months, while sufficient to capture most recurrences and functional changes, may not account for very late complications or re-triggers. A limitation of this study is the absence of consistently recorded operative time and the lack of a standardized patient satisfaction assessment. Another limitation of this study is the absence of a formal cost analysis. Although UGPR is presumed to be more cost-effective due to its outpatient applicability and avoidance of sutures or operating room resources, future studies should include structured economic evaluations to confirm this assumption.

## Figures and Tables

**Table 1:** Descriptive statistics on demographic and clinical variables in patients with trigger finger.

		Method		
	All Patients	USG (*n* = 75)	Open (*n* = 71)	*p*-Value
**Age** ^†^	58.3 ± 9.6	57.4 ± 10.1	59.2 ± 9.0	0.246 *
**Sex** ^‡^				
Male	37 (25.3)	18 (24.0)	19 (26.8)	0.847 **
Female	109 (74.7)	57 (76.0)	52 (73.2)	
**Finger** ^‡^				
1 Finger	67 (45.9)	44 (58.7)	23 (32.4)	**0.030** **
2 Finger	11 (7.5)	5 (6.7)	6 (8.5)	
3 Finger	25 (17.1)	9 (12.0)	16 (22.5)	
4 Finger	38 (26.0)	15 (20.0)	23 (32.4)	
5 Finger	5 (3.4)	2 (2.7)	3 (4.2)	
**Hand Direction** ^‡^				
Left	54 (37.0)	29 (38.7)	25 (35.2)	0.794 **
Right	92 (63.0)	46 (61.3)	46 (64.8)	
**Green Classification** ^‡^				
Stage 2	91 (62.3)	52 (69.3)	39 (54.9)	0.212 **
Stage 3	51 (34.9)	21 (28.0)	30 (42.3)	
Stage 4	4 (2.7)	2 (2.7)	2 (2.8)	
**Complication** *+* ^‡^	6 (4.1)	1 (1.3)	5 (7.0)	0.109 **
**Revision**, *+* ^‡^	4 (2.7)	4 (5.3)	0 (0.0)	0.120 **
	**Method**			
	**USG (*n* = 75)**	**Open (*n* = 71)**		***p*-Value**
**VAS**				
0	6.0 [4.0–8.0]	6.0 [4.0–8.0]		0.409
3	6.0 [4.0–9.0]	4.0 [3.0–7.0]		**<0.001**
1 Month	2.0 [0.0–5.0]	2.0 [0.0–4.0]		0.053
6 Month	0.0 [0.0–3.0]	0.0 [0.0–2.0]		0.420
12 Month	0.0 [0.0–1.0]	0.0 [0.0–1.0]		0.561
*p*-value **	**<0.001**	**<0.001**		

* *t*-test for independent samples. ** Pearson Chi-Square/Fisher’s Exact/Fisher Freeman Halton test.

**Table 2:** Intergroup and intragroup comparisons of DASH, NIRSCHL, and VAS scores in patients with trigger finger who underwent USG-guided percutaneous release and open surgery.

	Method		
	USG (*n* = 75)	Open (*n* = 71)	*p*-Value
**DASH**			
0	38.6 [25.0–61.3]	40.9 [25.0–61.3]	0.710
3	40.9 [25.0–61.3]	34.0 [20.4–59.0]	**<0.001**
1 Month	7.9 [4.5–43.1]	4.5 [2.2–11.3]	**<0.001**
6 Month	2.2 [0.0–4.5]	2.2 [0.0–4.5]	0.409
12 Month	0.0 [0.0–9.0]	0.0 [0.0–2.2]	0.297
*p*-value **	**<0.001**	**<0.001**	
	**Method**		
	**USG (*n* = 75)**	**Open (*n* = 71)**	***p*-Value**
**NİRSCHL**			
0	4.0 [2.0–6.0]	4.0 [2.0–6.0]	0.499
3	5.0 [3.0–7.0]	3.0 [2.0–5.0]	**<0.001**
1 Month	2.0 [0.0–5.0]	2.0 [0.0–5.0]	0.081
6 Month	0.0 [0.0–2.0]	0.0 [0.0–2.0]	0.409
12 Month	0.0 [0.0–1.0]	0.0 [0.0–1.0]	0.650
*p*-value **	**<0.001**	**<0.001**	

** Pearson Chi-Square/Fisher’s Exact/Fisher Freeman Halton test.

**Table 3:** Intergroup and intragroup comparisons of pinchmeter measurements in USG-guided percutaneous loosening and open excision cases in patients with trigger finger.

	Method		
	USG (*n* = 75)	Open (*n* = 71)	*p*-Value *
**Pinchmetre**			
0	4.2 [0.8–7.4]	3.3 [0.6–7.1]	0.059
12 Month	5.2 [1.1–9.1]	4.0 [1.1–9.3]	**0.008**
*p*-value **	**<0.001**	**<0.001**	

* Mann–Whitney U test. ** Wilcoxon test. Descriptive statistics were given as median [Min.–Max.] for numerical variables.

**Table 4:** Intergroup and intragroup comparisons of pulley thickness and flexor tendon thickness measurements at the metacarpophalangeal joint level in patients with trigger finger treated with USG-guided percutaneous release and open surgery.

	Method		
	USG (*n* = 75)	Open (*n* = 71)	*p*-Value *
**Pulley Thickness**			
0	1.4 [1.1–1.8]	1.3 [0.9–1.7]	<0.053
12 Month	0.4 [0.2–0.9]	-	-
*p*-value **	**<0.001**	-	
**Δ Pulley thickness**	−66.67 [−84.09–−40]	-	-
	**Method**		
	**USG (*n* = 75)**	**Open (*n* = 71)**	*p*-Value *
**MCP fleksör Tend**			
0	3.4 [2.7–5.0]	3.1 [2.6–4.1]	<0.001
12 Month	3.8 [2.8–6.3]	3.6 [2.8–4.5]	0.086
*p*-value **	**<0.001**	**<0.001**	
**Δ MCP flexor tendon**	7.14 [−21.62–85.29]	12.43 [−3.23–22.92]	**<0.001**

* Mann–Whitney U test. ** Wilcoxon test. Descriptive statistics were given as median [Min.–Max.] for numerical variables.

## Data Availability

The original contributions presented in this study are included in the article/Supplementary Materials. Further inquiries can be directed to the corresponding author.

## Supplementary Materials

The following supporting information can be downloaded at https://www.mdpi.com/article/10.3390/jcm14197064/s1: Figure S1: Release of A1 pulley with 18G needle under ultrasound guidance; Table S1: Outcomes at 12-Month Follow-up in Thumb-Only Cases; Table S2: Outcomes at 12-Month Follow-up in Non-Thumb Cases.

## Abbreviations

The following abbreviations are used in this manuscript:

UGPR	Ultrasound-guided percutaneous release
kg	Kilograms
ASHT	American Society of Hand Therapists
NPRS	Nirschl Phase Rating Scale
VAS	Visual Analog Scale
QuickDASH	Quick Disabilities of the Arm, Shoulder, and Hand
USG	Ultrasonographic
MCP	Metacarpophalangeal
