# Supplementary material for: Ultrasound-Guided Percutaneous Versus Open A1 Pulley Release for Trigger Finger: A Randomized Controlled Trial
Source: J Clin Med. 2025 Oct 7;14(19):7064. doi: 10.3390/jcm14197064 (PMC12525031; doi:10.3390/jcm14197064)
Supplement: Supplementary file 1 [file jcm-14-07064-s001.zip › jcm-3889199-supplementary.pdf]

Supplementary Table S1. Outcomes at 12-Month Follow-up in Thumb-Only Cases

Outcome	UGPR (n=44) Median [IQR]	Open (n=23) Median [IQR]	p-value
QuickDASH	0.0 [0.0–0.0]	0.0 [0.0–0.0]	0.443
VAS	0.0 [0.0–0.0]	0.0 [0.0–0.0]	1.000
Pinch Strength (kg)	7.0 [6.3–7.8]	7.2 [5.9–7.8]	1.000
MCP Tendon (mm)	3.5 [3.4–3.9]	3.3 [3.1–3.8]	**0.049**

Supplementary Table S2. Outcomes at 12-Month Follow-up in Non-Thumb Cases

Outcome	UGPR (n=31) Median [IQR]	Open (n=48) Median [IQR]	p-value
QuickDASH	0.0 [0.0–0.0]	0.0 [0.0–0.0]	0.638
VAS	0.0 [0.0–0.0]	0.0 [0.0–0.0]	0.316
Pinch Strength (kg)	3.1 [2.0–4.2]	2.8 [1.7–4.0]	0.551
MCP Tendon (mm)	4.0 [3.8–4.2]	3.6 [3.5–4.0]	**0.006**

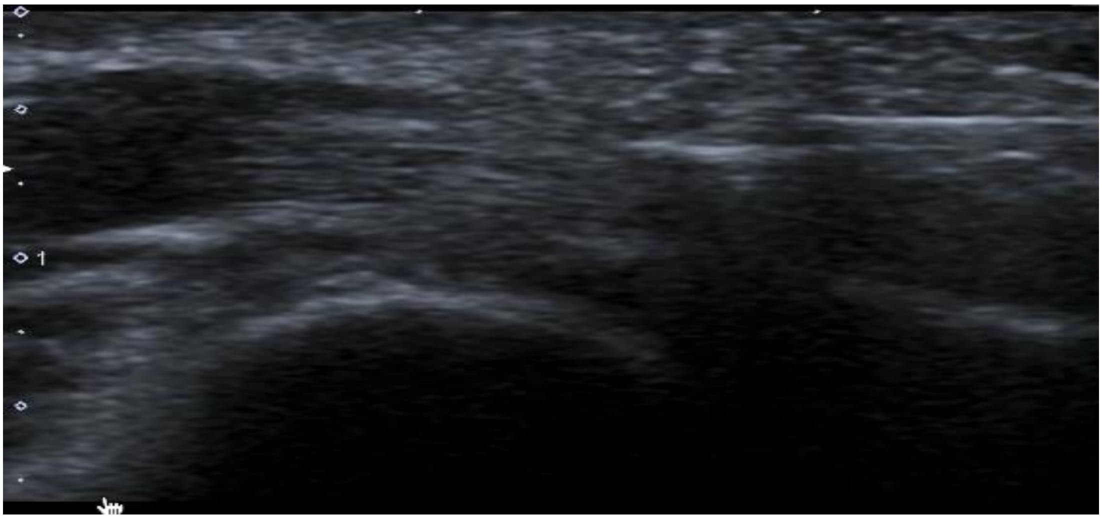

Figure S1. Release of A1 pulley with 18G needle under ultrasound guidance
